# Comparison of Three 3D Segmentation Software Tools for Hip Surgical Planning

**DOI:** 10.3390/s22145242

**Published:** 2022-07-13

**Authors:** Marco Mandolini, Agnese Brunzini, Giulia Facco, Alida Mazzoli, Archimede Forcellese, Antonio Gigante

**Affiliations:** 1 Department of Industrial Engineering and Mathematical Sciences, Università Politecnica delle Marche, Via Brecce Bianche 12, 60131 Ancona, Italy; a.brunzini@staff.univpm.it (A.B.); a.forcellese@staff.univpm.it (A.F.); 2 Dipartimento di Scienze Cliniche e Molecolari, Università Politecnica delle Marche, Via Tronto 10/a, Torrette di Ancona, 60126 Ancona, Italy; g.facco@pm.univpm.it (G.F.); a.p.gigante@staff.univpm.it (A.G.); 3 Department of Materials, Environmental Sciences and Urban Planning, Università Politecnica delle Marche, Via Brecce Bianche 12, 60131 Ancona, Italy; a.mazzoli@staff.univpm.it

**Keywords:** CT image segmentation, reverse engineering, software comparison, orthopaedics, hip surgery, bio-imaging, surgical planning

## Abstract

In hip arthroplasty, preoperative planning is fundamental to reaching a successful surgery. Nowadays, several software tools for computed tomography (CT) image processing are available. However, research studies comparing segmentation tools for hip surgery planning for patients affected by osteoarthritic diseases or osteoporotic fractures are still lacking. The present work compares three different software from the geometric, dimensional, and usability perspectives to identify the best three-dimensional (3D) modelling tool for the reconstruction of pathological femoral heads. Syngo.via Frontier (by Siemens Healthcare) is a medical image reading and post-processing software that allows low-skilled operators to produce prototypes. Materialise (by Mimics) is a commercial medical modelling software. 3D Slicer (by slicer.org) is an open-source development platform used in medical and biomedical fields. The 3D models reconstructed starting from the in vivo CT images of the pathological femoral head are compared with the geometries obtained from the laser scan of the in vitro bony specimens. The results show that Mimics and 3D Slicer are better for dimensional and geometric accuracy in the 3D reconstruction, while syngo.via Frontier is the easiest to use in the hospital setting.

## 1. Introduction and Literature Review

Preoperative planning is a fundamental step toward a successful surgery, especially for hip arthroplasty. Indeed, when a hip replacement is needed, inadequate prosthesis fitting, related loosening of the implants, and poorly performed surgery may result in severe implications for the patient [1,2]. Therefore, computed tomography (CT) and anatomical 3D model reconstruction are often performed before arthroplasty to identify the implant that best replicates the patient’s joint anatomy [3,4]. However, due to the surrounding tissues, bone deterioration, and remodelling, the correct interpretation of the bone CT and related 3D model reconstruction may be difficult and inaccurate [5]. The complexity of the anatomical 3D modelling further increases in the case of osteoarthritic, congenital, osteonecrosis, osteoporotic, and post-traumatic diseases [6,7]. Indeed, congenital disorders such as developmental dysplasia of the hip, Legg–Perthes–Calvé disease, and slipped capital femoral epiphysis cause remodelling of the hip joint resulting in an alteration of the normal anatomy of the joint [8]. Bone cell death, which leads to the collapse of articular cartilage, is the leading cause of femoral head osteonecrosis. Osteoarthritis is a degenerative disease that affects the joints. It is characterised by the loss of articular cartilage associated with subchondral bone sclerosis and the production of osteophytes and geodes [9]. The leading causes of secondary osteoarthritis include osteonecrosis and congenital and post-traumatic hip diseases [10]. Even osteoporosis is characterised by changes in bone biologic material and structural distraction with the consequent reduction in the resistance of bone tissue that predisposes to an increased risk of fractures [11]. In all these clinical situations, an anatomical 3D model results in being helpful in surgical planning [12,13] and communication with the patients [14,15]. Moreover, it has been demonstrated that the anatomical 3D model helps reduce surgery duration [16,17] and intra-operative blood loss, allowing better surgical outcomes [18].

The segmentation process is the basis of anatomical 3D reconstruction. It separates and renders the regions of interest (ROI) from the surrounding tissues and structures. However, only cortical bone has the high density required to differentiate it from adjacent soft tissues. In contrast, the density of porous bones differs only slightly from that of the soft tissues. This phenomenon is mainly observable in elderly patients, i.e., a population that frequently suffers from bone weakness. To perform proper bone identification, it is necessary to achieve a substantial contrast between bones and soft tissues [19]. Segmentation can be performed manually, semi-automatically, or automatically. While the manual approach is user-dependent and time-consuming, the automatic and semi-automatic methods are faster and computer-aided [20]. However, several issues arise even with automatic and semi-automatic methods due to the technical and computational complexity of the 3D image reconstruction and segmentation. New, advanced CT reconstruction algorithms are constantly being developed and commercially deployed to improve the classification and recognition of patterns inside biomedical images. The most used and most efficient ones are based on deep learning convolutional neural networks. Several studies have investigated their benefits, weaknesses, and their impact on the reconstructed image quality [21,22,23]. Multiple segmentation methods could be used together on a single 3D model to eliminate minor deficiencies intrinsic to every algorithm [24].

Nowadays, several software tools for processing DICOM (Digital Imaging COmmunications in Medicine) files are available on the market and freely online. Mimics, Medviso, 3D Slicer, ITK-SNAP, MeVisLab, and ImageJ can be included among the most used and best known segmentation software [24,25]. These tools can be divided into three categories: CT embedded, high-end licensed, and open source software. They present different segmentation algorithms, reconstruction accuracy, levels of usability, and, of course, commercial costs. Many studies in the scientific literature have tried to compare segmentation software, focusing on the reconstruction of specific anatomical districts or related to particular kinds of patients or diseases; several examples are described hereafter.

A specific field of interest for the segmentation tools is the 3D reconstruction of organs such as the lungs [26,27,28]. However, the present work focuses on the segmentation of bone structures. Considering maxillofacial surgery, several authors assessed and compared the segmentation tools for the 3D modelling of the skull and mandible. Through the comparison between the commercial software Mimics (used as the gold standard) and the open source Medical Imaging Interaction Toolkit (MITK) [29], InVesalius [20,30], ITK-Snap, 3D Slicer, and the on the market Dolphin 3D [20], the authors found out that the 3D models produced using Mimics and the other software were comparable [20,29,30]. Therefore, they suggested using open source software for preoperative planning to minimise the operational cost [29,30]. Wallner et al. (2018) assessed the accuracy of a license-free segmentation algorithm using the MeVisLab software as ground truth. In this case, the results suggested that the proposed open source software could be used with high accuracy to segment the craniomaxillofacial complex [31]. The parameters used for the comparisons include the Hausdorff distance [29,30,31], Wilcoxon signed rank test [29], Dice score [30,31], the segmentation volume [20,31], time, and the number of voxels [31]. A general review of the metrics for evaluating 3D medical image segmentation is available in Taha et al. [32]. Other performance parameters considered for the software evaluation are the time needed to complete the segmentation, ease of use, algorithm precision [33], automatisation degree, 3D visualisation, image registration tools, tractography tools, supported OS, and potential plugins [25]. In [25], the authors compared fourteen software tools (twelve free source and two commercial) to obtain patient-specific 3D models of the pelvic region of children from magnetic resonance images (MRI). In [33], the authors used three open source software to segment CT images of a vertebra.

Segmentation software comparison for hip surgery planning, for patients affected by osteoarthritic diseases or osteoporotic fractures, is still scarce in the literature. However, the authors in [34] investigated the use of different segmentation algorithms for the 3D reconstruction of a human cadaver femur, divided into four areas: “*neck and greater trochanter*”, “*proximal metaphysis*”, “*diaphysis*”, and “*distal metaphysis*”. The deviation analysis was carried out between the femur models reconstructed from the CT images and those obtained from the optical 3D scanning of the femur. The results showed that the average deviation of CT-based models from the scan models is very low. However, the highest deviation error was found in the “neck and greater trochanter” area [34]. This area is essential for the planning of hip surgery. It is even the most affected area in osteoarthritic disease and is thus the most difficult to segment correctly. That is why the present work focuses on femoral heads, leaving behind the other hip sections.

Every surgeon should trust CT-based models, obtained by in vivo bone, to reach good surgical outcomes. For this reason, in contrast with the previously cited research, which was performed only on cadaver femurs, the present work considers the CT images of in vivo femoral heads with the surrounding soft tissues. This paper aims to compare three different software tools in the 3D modelling of pathological in vivo femoral heads from CT data:

- *Mimics v.12.11* (*by Materialise, Leuven, Belgium*): commercial medical 3D image-based engineering software that efficiently takes images to 3D models;
- *3D Slicer v.4.10.2* (*by Slicer Community, www.slicer.org*): open source software to solve advanced image computing challenges and a development platform for medical and biomedical applications;
- *Syngo.via Frontier 3D printing v.1.2.0* (*by Siemens Healthcare GmbH, Erlangen, Germany*): medical image reading and post-processing software that allows low-skilled operators to produce prototypes.

The presented comparison wants to establish the best image segmentation solution for hospital facilities. On the one hand, geometrical and dimensional accuracy is mandatory for estimating the quality of 3D segmented models. However, in a clinical environment, this is not enough. Easy-to-use devices should support doctors and technicians. Hence, software usability is another metric that must be evaluated in this context.

Thus, the comparison concerns three aspects:

- *Geometric quality*: geometric accuracy of the segmented femur heads;
- *Dimensional quality*: dimensional accuracy of femur heads’ measurements (vertical and horizontal diameters);
- *Usability*: user experience during the overall segmentation process for preoperative hip planning.

To the authors’ knowledge, this is the first paper whose software comparison is based on in vivo femoral head images. This research will help hospitals and clinicians improve hip surgery preoperative planning.

## 2. Materials and Methods

**Figure 1:**
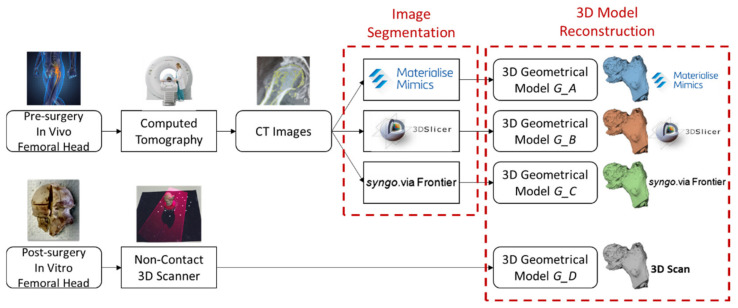
Pipeline’s part 1: Methodology for obtaining the 3D anatomical models to be compared (from the pre-surgery in vivo femoral heads and post-surgery in vitro bony specimens).

**Figure 2:**
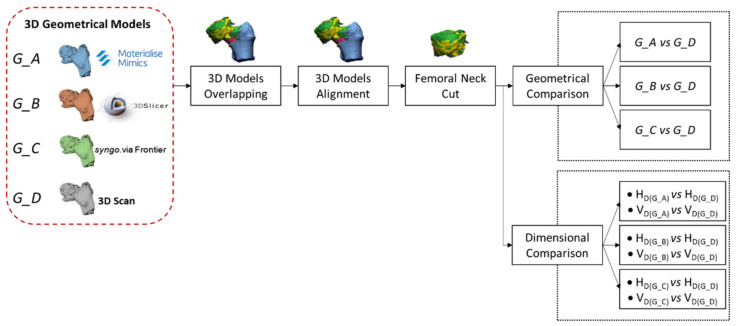
Pipeline’s part 2: 3D models comparison workflow. H_D_ and V_D_ are, respectively, the horizontal and vertical diameters of the femoral head.

Figure 1 and Figure 2 show the pipeline of the proposed methodology to compare the 3D models of pathological femoral heads. The 3D models obtained from three different segmentation software from the pre-surgery CT images are compared against the reference models reconstructed from a laser scan of the in vitro bony specimens. This methodology analyses the reliability of the 3D anatomical models reconstructed from the CT images using software with different technological characteristics by comparing them against the reference gold standard.

Figure 1 shows the first part of the pipeline. The computed tomography of the in vivo femoral head is acquired before the patient’s surgery. Then, the CT image segmentation occurs with three different software, Mimics, 3D Slicer, and syngo.via Frontier. This procedure results in three geometrical models of the same femoral head, respectively, the *G_A* (3D model reconstructed using Mimics), *G_B* (3D model reconstructed using 3D Slicer), and *G_C* (3D model reconstructed using syngo.via Frontier) geometries. Therefore, different algorithms reconstruct these models by distinguishing between the bone region and the surrounding tissues.

Then, the reference digital 3D model is realised. It considers the digitalisation, through a non-contact 3D laser scan, of the in vitro bony specimen collected after the patient had undergone hip replacement surgery. The obtained 3D model (*G_D*) is considered the reference since the 3D laser scanner is more accurate than the computed tomography. The surface of the cleaned bone is acquired without any adjacent tissue or material. The specimen is scanned outside the container with the preservative solution.

The reconstructed 3D geometrical models are the input of the second part of the pipeline (Figure 2).

**Figure 3:**
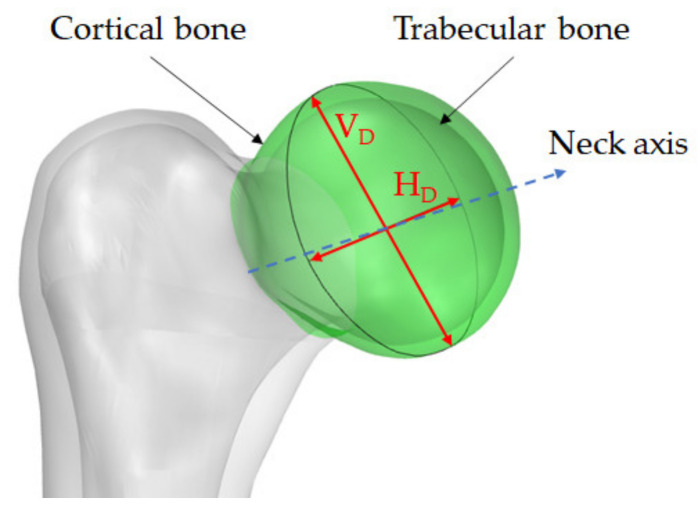
Horizontal (H_D_) and vertical (V_D_) diameters of a femoral head.

Since the 3D geometries are reconstructed through software with different reference systems, the models could not have the same orientation. For this reason, the first step consists of a manual 3D model overlapping (three-point method), assuring the same direction. Then, in the second step, the four 3D geometries must be aligned among themselves in the most precise and accurate way. This objective can be accomplished through two sub-steps: the best fit alignment algorithm should be run based on points selection after a manual alignment. The best fit alignment is possible because the femur head is not a perfect sphere [35], as illustrated in Figure 3. The femur head shape is mainly defined by the vertical (V_D_) and horizontal (H_D_) diameters. Vertical diameter is the “*maximal diameter of the femoral head taken in the vertical plane that passes through the axis of the neck*” [35]. Horizontal diameter is the “*maximal diameter of the femoral head taken in the horizontal plane perpendicular to the vertical diameter of the head*” [35]. Their mean difference is approximately 0.48 mm in men and 1.37 in females [36], with V_D_ > H_D_. Since the best fit is used to optimize the alignment between the geometries, according to the deviations mentioned above on the femur head diameters, a maximum allowed displacement is set to 1.0 mm. This value guarantees only a fine geometry adjustment without compromising the original manual alignment.

The femoral neck must be cut to avoid mismatches and focus the analysis on the femoral head. The neck trim after the models’ alignment makes the cut consistent for all the geometrical models. At this point, the comparison can be split into two other evaluations: the geometric and dimensional quality assessments. For the geometric comparison, *G_D* geometry is set as the reference model and, in turn, *G_A*, *G_B*, and *G_C* models as test. Results refer to the point-to-point surface distance (signed Euclidean distance) between the test and the reference models in terms of average and standard deviation.

It is noted that models reconstructed via CT segmentation tools contain both the femur head’s cortical (external) and trabecular (internal) structures. In contrast, the reference geometry obtained via reverse engineering has only the outer femur head shape (cortical). A maximum distance value was set to 2.0 mm to evaluate deviations only between external bodies, considering that the average cortical thickness in this region is around 1.3 ± 0.2 mm [37]. In addition, the maximum allowed error in surgical planning is about 2 mm.

Beyond the signed Euclidean distance, the authors evaluated the vertical (V_D_) and horizontal (H_D_) diameters of the femoral heads (Figure 3). Through this dimensional evaluation, it is possible to mimic the measurements in practice of surgeons, who measure the vertical and horizontal diameters of the femoral head through a specific calliper. The observed dimensions were obtained using the minimum bounding box algorithm applied to the four geometries (*G_A*, *G_B*, *G_C*, and *G_D*). Since the four geometries have been cut at the end of the neck, the relative bounding boxes are aligned with the neck axis (per V_D_ definition). The maximum and minimum dimensions of the boxes measured on the plane perpendicular to the neck axis are associated with the vertical and horizontal diameters.

Lastly, the usability of the used software tools must be assessed. As for the geometrical and dimensional analyses, even the usability was conceived as a comparison assessment. Seven evaluation objectives were defined and weighted by a focus group of experts. Five objectives were retrieved from [25,33]. Two were specified by the authors (training time and cost):

- *Automatisation degree*: amount of manual interaction required by the user [25].
- *Segmentation time*: time required for the segmentation [25].
- *3D visualisation*: ability to represent a 3D model realistically [25].
- *Supported Operative System* (*OS*): supported operative systems [25].
- *Potential extension* (*plugins*): ability of the software tools to be freely extended by add-ons or plugins [33].
- *Training time*: time required to start using the tool.
- *Cost*: license price.

First, the focus group expressed n-weights (*Weight_n_*) for each evaluation objective (*n*). It then defined the scores (*Score_n_*) for each metric and software tool on a three-point scale. The total score for each software is computed through the weighted arithmetic mean equation (Equation (1))

(1) $$Total\;score=\frac{Weight_{n}*Score_{n}}{\sum_{n}Weight_{n}}$$

## 3. Case Study

This section presents the application of the above-mentioned methodology to compare the software tools used for femur head image segmentation for hip surgical planning.

### 3.1. Participants

Since this study wanted to evaluate segmentation software tools in a clinical environment, patients were enrolled to represent a specific population requesting hip surgical preplanning. Thus, the research involved ten patients with different pathologies (e.g., primitive or secondary coxarthrosis or hip fractures) and femur head shapes who needed total hip arthroplasty. Osteoarthritic, congenital, osteonecrosis, osteoporotic, and post-traumatic diseases are pathologies that must be managed to robustly evaluate segmentation software in case of anatomical 3D modelling complexity.

Subjects who needed hip revision surgery and patients not subjected to a preoperative CT of the pelvis were excluded. Ten patients signed the informed consent to use the preoperative CT images of the pelvis and the bony specimen derived from surgery (femoral head) for scientific research purposes. The ten patients underwent a preoperative CT scan of the pelvis using a CT machine with 120 kV and 0.625 slice thickness. Their demographic data and information related to pathology and CT images are described in Table 1.

Bony specimens of femoral heads were cleaned from blood and placed in containers with formalin. The results refer to eight patients because two of them were discarded based on the following exclusion criteria:

- The presence of large geodes required massive intervention by the operator to reconstruct the 3D model. This condition caused the inevitable introduction of errors concerning the 3D model rebuilt from the scan of the in vitro bony specimen.
- The deterioration of the bony specimen (the femur head was poorly preserved) made the scan impossible to perform.

### 3.2. Procedure

An expert and trained operator produced the 3D digital models of the pathological femoral heads. The operator had good experience with the three software tools. He had already used them in more than ten case studies per software to reconstruct pathological bones.

The three segmentation software tools can be briefly described as:

- *Materialise Mimics*: a commercial medical modelling software that allows interfacing between CT data and a computer-aided design (CAD) or solid free-form fabrication (SFF) systems.
- *3D Slicer*: a development platform to quickly and freely build and deploy custom research and commercial product solutions.
- *Siemens syngo.via Frontier*: a medical image reading and post-processing software that quickly creates prototypes regardless of expertise level.

Overall, 3D anatomy reconstruction required the initial segmentation of the anatomical bone structures via a CT scan. CT is unable to detect cartilage and the remaining soft tissues. However, articular cartilage was poorly represented in these patients. The resultant 2D image slices were stored in DICOM format. The CT images were then used as the primary data for reconstructing the 3D models. The bony areas were extracted from each slice to obtain 3D anatomy images. Because pathological hips did not have joint space between the head and acetabulum in some places, only one pixel was manually removed to stay below the imaging resolution threshold in each 2D slice. The 3D visual models were then acquired by stacking the segmented slices; the transformation from the sliced images to the STL (standard triangulation language) format employed the marching cubes algorithm. The 3D visual models of the anatomy were converted, and STL format surface models were created. Therefore, the 3D model of the femoral head was reconstructed for each patient with the three software Mimics, 3D Slicer, and syngo.via Frontier following the same four steps:

- DICOM files from pelvis CT were imported. Br 64, a high-resolution CT convolution kernel, was used for reconstruction images.
- Only the femoral head was included in the region of interest (ROI). Greater and lesser trochanters were excluded.
- The bone identification was made by a threshold segmentation process selecting over 200 Hounsfield Units (HU). To separate the femoral head from the acetabulum, manual segmentation was performed by removing only one pixel to remain below the imaging resolution threshold in each 2D slice. Manual segmentation was conducted to remove all the pixels that do not belong to the femoral head in each 2D slice.
- The STL file of the 3D model was exported (3D models: *G_A* by Mimics, *G_B* by 3D Slicer, and *G_C* by syngo.via Frontier).

The DICOM images were analysed using three different software tools, dividing the patients into three groups. The first three-patient group analysis started with the syngo.via Frontier tool. In contrast, the second group analysis (other three patients) began with 3D Slicer, and the third group analysis (four patients) started with Mimics. This procedure was defined to avoid eventual bias related to the consecutive use of the software tools, always in the same sequence. Indeed, this anti-bias procedure aimed to prevent the operator from remembering the geometrical bone structures, thus having the best results with the last software.

*G_A* model segmentation required about 35 min (from 30 to 40 min), *G_B* about 45 min (from 40 to 50 min), and *G_C* about 40 min (from 30 to 50 min).

Subsequently, the reference 3D models were reconstructed after surgery, scanning the in vitro femoral head specimens. The 3D laser Scanner *Range v.7* (by Konica Minolta, Tokyo, Japan) was chosen to digitise the cleaned bony samples. It was selected for its accuracy to produce the reference 3D models of the femoral heads. Indeed, the 3D laser scanner’s accuracy (40 µm) is higher than that of the CT machine (1250 µm). The comparison between the different 3D models was accomplished using the 3D point clouds and mesh processing software *CloudCompare v.2.10.2* (open source, by www.cloudcompare.org). The comparison procedure proposed in Figure 2 was followed in this case study. In detail:

- *G_A*, *G_B*, and *G_C* geometries were overlapped in *Rhinoceros 3D v.6* (by Robert McNeel and Associates, Seattle, USA) to have them in the same orientation. *G_B* geometry was rotated at 180° around the z-axis to have it superimposed on the *G_A* and *G_C* geometries (already correctly oriented).
- *G_B* geometry was exported in STL format.
- *G_A*, *G_B*, and *G_C* geometries, which are already overlapped, were imported into *Geomagic Design X* (by 3D Systems, Rock Hill, USA).
- *G_A*, *G_B*, *G_C*, and *G_D* geometries were then aligned in *Geomagic Design X* through a two steps procedure:
  ○ The manual alignment of geometry *G_D* concerning geometries *G_A*, *G_B*, and *G_C* (already aligned with each other), with three reference points. In particular, the alignment was conducted between geometry *G_D* and *G_A* (the one with the best external surface).
  ○ The automatic alignment (best fit algorithm) of *G_D* geometry concerning the *G_A*, *G_B*, and *G_C* geometries (as for the previous step, the *G_A* geometry was chosen for the alignment).
  ○ The femoral necks were cut to leave only the femoral heads. In this way, the cut consistently occurs for all the geometries.
  ○ The four geometries were exported in STL format.
- Geometric quality evaluation:
  ○ The four geometries were imported into *CloudCompare*.
  ○ The comparison was performed by setting one of the geometries from CT as the test (*G_A*, *G_B*, *G_C*) and the *G_D* geometry as the reference. The maximum deviation was set at 2 mm.
  ○ The comparison was repeated for the other two segmented models.
  ○ The results of the comparison were exported as mean values and standard deviation.
- Dimensional quality evaluation:
  ○ The four geometries were imported into *Rhinoceros 3D*.
  ○ A Phyton script was executed for computing the minimum bounding box for all four geometries.
  ○ The maximum and minimum bounding box dimensions measured on a plane perpendicular to the femoral neck were, respectively, assigned to the vertical and horizontal diameters.
  ○ The comparison was performed by setting the diameters measured on the *G_D* geometry as the reference and the other diameters computed on *G_A*, *G_B*, and *G_C* geometries as the test.

## 4. Results

Three types of results were obtained in the present work and are presented as follows:

- *Geometric quality*: this is the geometric deviation between the reference and test geometries of the femoral head. This type of result helps evaluate how much the segmentation tools can precisely reconstruct the external surface of the femoral heads.
- *Dimensional quality*: this is the femoral head diameters’ deviation between the reference and test geometries. This result allows surgeons to evaluate how precisely the segmentation tools can catch the femoral head dimensions.
- *Usability*: this evaluation refers to several metrics (e.g., automatisation degree, segmentation time, training time), which are helpful for surgeons to evaluate the segmentation tools globally.

### 4.1. Geometric Quality

**Figure 4:**
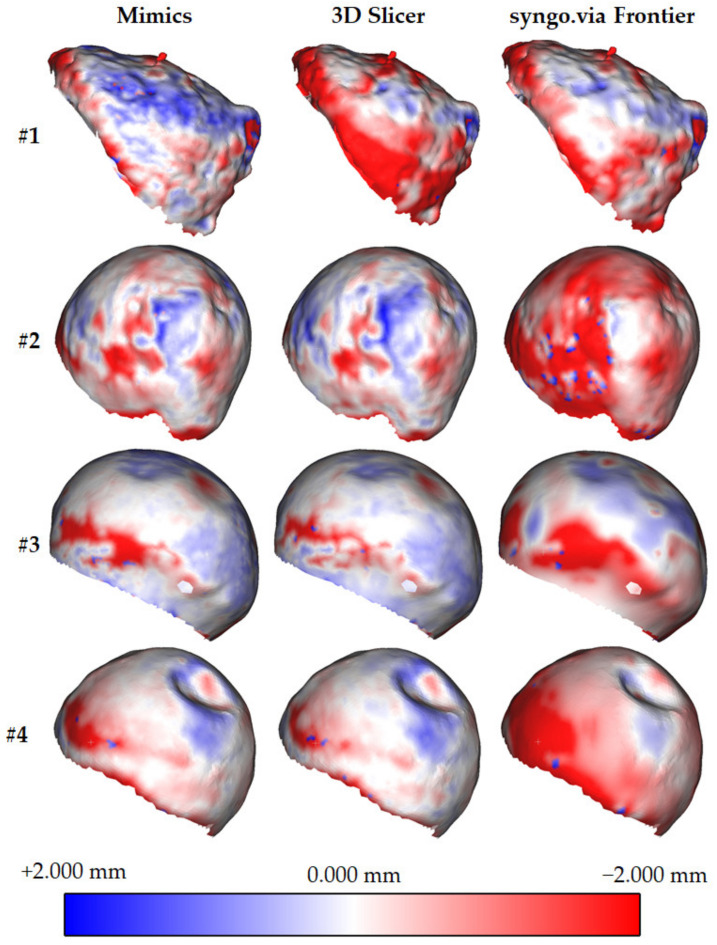
Signed Euclidean distance between the reference (*G_D*) and test (*G_A*, *G_B*, and *G_C*) geometries for patients #1, #2, #3, and #4. The deviation is represented in the reference geometry. Red: −2.000 mm deviation. White: 0.000 mm deviation. Blue: +2.000 mm deviation.

**Figure 5:**
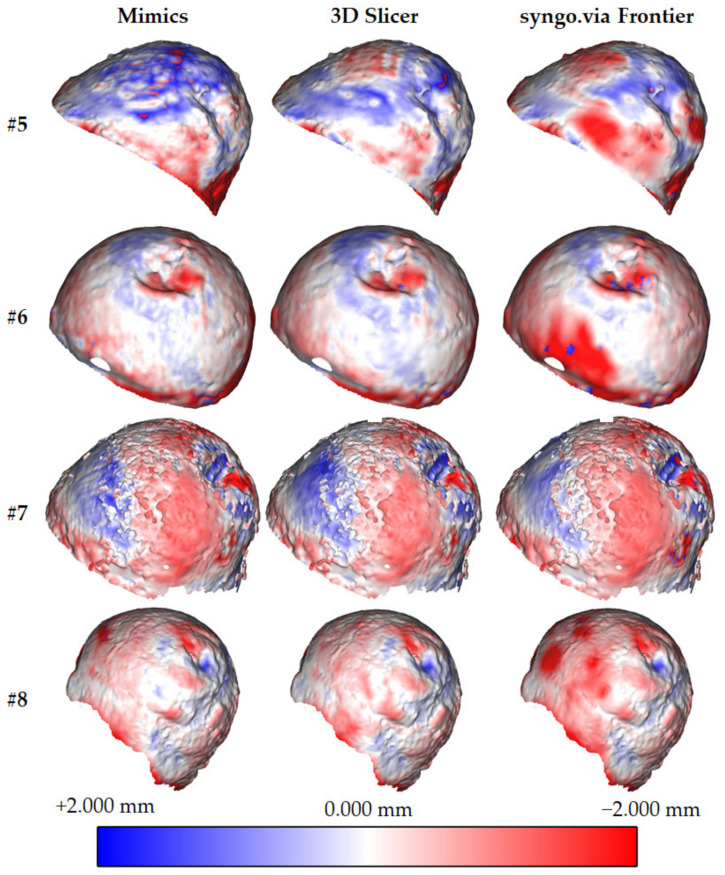
Signed Euclidean distance between the reference (*G_D*) and test (*G_A*, *G_B*, and *G_C*) geometries for patients #5, #6, #7, and #8. The deviation is represented in the reference geometry. Red: −2.000 mm deviation. White: 0.000 mm deviation. Blue: +2.000 mm deviation.

**Figure 6:**
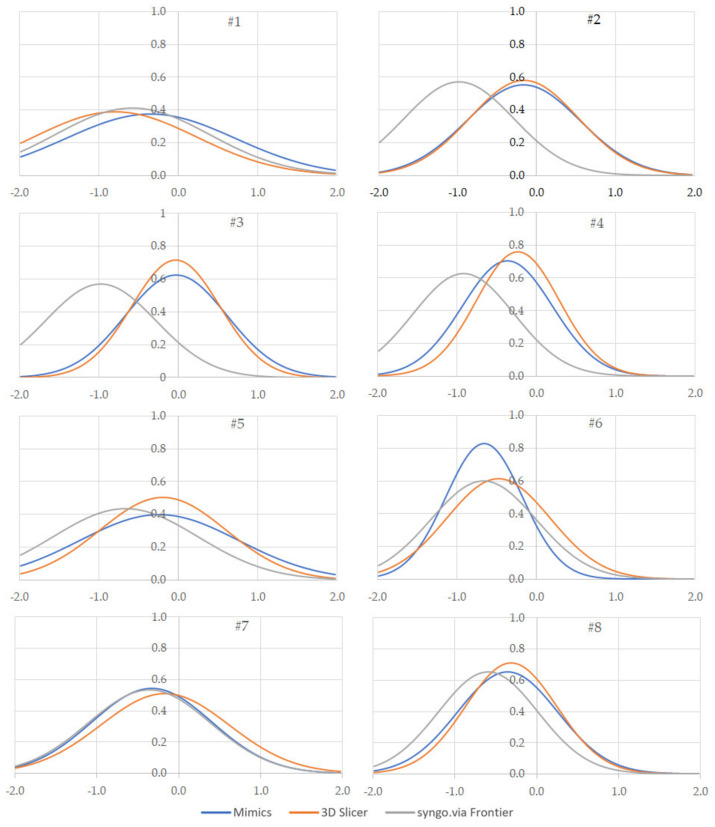
Signed Euclidean distance between the reference (*G_D*) and test (*G_A*, *G_B*, and *G_C*) geometries.

Following the previously described procedure, the authors evaluated the signed Euclidean distance (Figure 4, Figure 5 and Figure 6, Table 2) and average distances between the reference (*G_D*) and the test (*G_A*, *C_B*, and *G_C*) geometries for each patient. Such indicators were selected from twenty metrics used to evaluate 3D medical image segmentation [32]. This selection was made considering the segmentation requirements of this work, that is:

1. *The low segmentation quality*: often, the patient’s pathologies in this work determined low-quality segmented volumes.
2. *The high complex boundaries and presence of outliers*: this requirement results from the previous one. Since the external surfaces of the segmented volumes are irregular, outliers may exist.
3. *The high importance of the contour*: the contour is relevant because it evaluates the hip implant dimensions.
4. *The low relevance of the volume*: the external surfaces of the segmented volumes are often non-continuous. A robust volume evaluation was not possible.

Figure 4 and Figure 5 show the colour maps for all the patients. To uniformly evaluate the results, deviations are always represented on the reference geometries. Each figure contains a colour scale map (red: maximum negative deviation (−2 mm); blue: maximum positive deviation (+2 mm); white: no deviation).

Signed distance was considered for evaluating both the deviation and direction. The first result is required for assessing the best software tool (in terms of accuracy). The second is necessary to understand whether the test geometries are bigger or smaller than the reference. For each comparison, the authors evaluated the average distance and relative standard deviation (Table 2).

Average and standard deviations were used to create graphs (one for each patient) plotting the distance distribution for each software tool (Figure 6). Such graphs allow a quick comparison of the software tools used to rebuild the femoral head geometries.

### 4.2. Dimensional Quality

In addition to the signed Euclidean distance, Table 3 presents the horizontal (H_D_) and vertical (V_D_) diameters of the femur heads taken on the different segmented geometries. The mean values between H_D_ and V_D_ for each test geometry (*G_A*, *G_B*, and *G_C*) are compared to the reference one to evaluate the deviations (Table 4). These results are required to evaluate the performance of the segmentation tools in assessing the implant dimensions for knee surgery.

### 4.3. Usability

The third set of results (Table 5) was defined considering the seven usability evaluation objectives. A single focus group (first limitation of this work) of one technician involved in the CT images’ segmentation, one surgeon, two PhD students, and two researchers on biomedical-related topics was set to define the weights for each objective (from one to ten) and the values for each software and goal. First, the focus group expressed weights. “*Automatisation degree*” is the most crucial criterion because CT scans should be rapidly elaborated, and the result should not depend on the technician. To achieve these goals, it is significant to employ automatic tools. “*Segmentation time*”, “*Training time*”, and “*Cost*” are also crucial because, in hospitals, segmentation tools need to be used by clinicians without an engineering background.

Furthermore, the deployment cost (CAPEX—CApital EXPenditure, training, and license cost for software) must be as low as possible to limit the impact on the national health system. “*3D visualization*” and “*Supported Operative System (OS)*” are not as important as the previous criteria because, nowadays, segmentation tools have good rendering characteristics, and Microsoft Windows Operative System is widespread. “*Potential extension (plugins)*” is marginal because 3D geometries segmented by the software are enough to evaluate dimensions and take decisions (no other functionalities are required).

Then, the focus group members defined (and agreed all together) their evaluations, which were converted into numerical scores (1: the worst software, 3: the best software). A three-point scale was used because the project goal was to compare the tools (an absolute score was not required). Ex aequo scores were assigned for those tools with the same performance for a specific criterion. At last, the weights and scores allowed the authors to rank the segmentation tools.

## 5. Discussion

The results discussion reflects the three types of results obtained and presented in the previous section.

Concerning the geometric quality, Table 2 allows the observation that the three software tools tend to reconstruct a smaller geometry than the actual one. The mean signed Euclidean distance is about 0.3 mm for Mimics and 3D Slicer, and 0.7 mm for syngo.via Frontier. This conclusion is aligned with the test performed in [34]. The authors compared the geometries obtained by employing four segmentation tools (i.e., Mimics, Amira, YaDiv, and Fiji-Medtool) with that achieved from a 3D optical scanner (GOM ATOS III). They found an average deviation of −0.72 (negative) and +0.66 mm (positive) with an average standard deviation of 0.63 mm. Mimics was the software with the lowest deviation. Measurements were made on the area of specific femur cross-sections.

This conclusion is also recognisable by analysing Table 4 (deviations in the femoral head diameters). Additionally, in this case, the three software tools tend to generate smaller femoral heads, the diameters of which are, on average, approximately 0.6 mm less for Mimics and 3D Slicer and 1.5 mm for syngo.via Frontier. It is noted that, with regards to the diameters, for two patients, Mimics and 3D Slicer estimated femoral heads with bigger diameters. In conclusion, the overall negative deviation should be considered in hip surgeries when the implant size is selected according to the 3D model reconstructed from CT images using the segmentation software tools.

Another conclusion can be drawn by comparing the segmentation quality of the software tools. Table 2 and Figure 6 show that 3D Slicer approximately performs similar to Mimics, with deviations, respectively, of −0.299 vs. −0.314 mm (standard deviation of 0.697 vs. 0.727 mm). Syngo.via Frontier exhibited a more significant geometric difference and standard deviation (respectively, 0.640 and 0.757 mm) due to the irregular surfaces of the segmented geometries. This conclusion is coherent with that drafted in [20], where a mandible was used as a test. Using Mimics and 3DSlicer for measuring the volume of mandibles, the deviations with a fully manual segmented geometry were 40.85 and 40.58 mm^3^, respectively. Such tools outperformed other solutions such as Dolphin 3D and InVesalius.

Furthermore, the results from [20] can be used to validate the robustness of our conclusions. In this study, eight patients who are not relevant for statistical analysis (this is the most significant limitation of this work) were considered. Nevertheless, for the dimensional quality evaluation (deviations for horizontal and vertical diameters) our results have a standard deviation between 1.0% and 3.4%, which is comparable with [20], where a greater number of patients (i.e., twenty) were taken. In the reference publication, the standard deviation is between 1.9% and 5.1%. Even if a more significant number of cases was convenient, our conclusions could be robust enough.

From Table 2, and Figure 4 and Figure 6, it is possible to evaluate that syngo.via Frontier generated femoral heads with irregular external surfaces, characterised by many holes. In Figure 4 and Figure 6, it is possible to observe that the maximum (negative) distance between the test and reference geometries is located in the region between the femoral head and neck. The reason could be linked to the highest curvature values in that region.

Concerning the robustness of the tools, from the geometric and dimensional evaluation (Table 2 and Table 4), globally, Mimics is the best (with regards to standard deviation). Concerning the geometric deviation, 3D Slicer exhibits, approximately, a standard deviation and mean absolute deviation similar to Mimics. Still, 3D Slicer behaves in a worse way for the dimensional variations (it is even worse than syngo.via Frontier), as visible in Table 4.

Another conclusion worth being drawn concerns patient #1 (surface collapse-geodes). The femoral head geometry, in this case, was entirely different from the other seven patients. Mimics was much better for this femoral head than the other two software with regards to the geometric and dimensional evaluations.

Concerning the usability evaluation, the authors’ findings contradict the geometric and dimensional quality (Table 5). Here, syngo.via Frontier outperforms Mimics and 3D Slicer. Syngo.via Frontier was much easier to use with a shorter learning curve since it has fewer functions and a more intuitive graphical interface.

Moreover, it is faster to use thanks to the direct connection with the DICOM database. Despite that, the accuracy of the geometrical prototype creation was lower than in other software such as Mimics. Accuracy is essential to implant the correct prosthetic components in osteotomies and more complex positions. While using syngo.via Frontier, surgeons must consider the possible accuracy errors during the operative planning to prevent any unforeseen errors.

Nevertheless, accuracy is not crucial for learning or communicating with patients. Regarding usability, 3D Slicer was the most difficult to use but with the significant advantage of being free. In contrast to the expensive high-end software, the open source software, such as 3D Slicer, must be considered, especially in small professional realities. That is why open source software can be a valuable tool with a competitive accuracy level despite being less user friendly and requiring longer times for model production.

The main limitation of this study is related to the small sample of participants. This set should be doubled in the future to obtain more robust results (as in [20,24,26]). Furthermore, the number of focus groups that evaluated the software tools should be extended. Additional evaluation units should be established in other hospitals (distributed in different countries) to understand if results are influenced by testers’ attitudes, backgrounds, and expertise. Other segmentation software tools (e.g., Medviso, ITK-SNAP, MeVisLab, ImageJ, InVesalius, and Dolphin) could be compared following the same methodology proposed in this study.

## 6. Conclusions

The processing of tomographic images for 3D models’ reconstruction can be arduous from a procedural point of view, especially for patients with pathologies that alter the morphology of hard and soft tissues. Therefore, in a clinical context, it is essential to understand which software best fits the surgeon’s expectations from different perspectives: the geometric and dimensional quality of the 3D model reconstruction and the usability of the segmentation tools.

This work precisely analysed three different categories of software for CT images’ segmentation (the high-end Materialise Mimics, the open source 3D Slicer, and the CT embedded Siemens syngo.via Frontier). Segmented 3D models were compared with a reference model obtained by the laser scan of the in vitro bony specimen.

The main results show that Mimics and 3D Slicer are better for the geometrical and dimensional accuracy in the 3D reconstruction of femur heads. The observed absolute average deviations (geometrical accuracy) are 0.299 mm (3D Slicer), 0.353 mm (Mimics), and 0.757 mm (syngo.via Frontier). The absolute percentage deviations in measuring the horizontal and vertical femur head diameters (dimensional accuracy) are 1.0% (Mimics), 1.4% (syngo.via Frontier), and 2.4% (3D Slicer).

At the same time, syngo.via Frontier is the easiest to use in the hospital setting with a high automatisation degree and requiring a low training time. Considering the “Automatisation degree”, “Segmentation time”, “Training time”, “Cost”, “3D visualization”, “Supported Operative System”, and “Potential extension (plugins)” usability criteria, a focus group with six participants gave scores of 2.28, 2.00, and 1.80, respectively, for syngo.via Frontier, 3D Slicer, and Mimics.

Future work will involve a more significant number of patients with osteoarthritic hips, allowing a more robust statistical analysis and characterisation of the software tools. It is expected to achieve at least 20 cases, similar to other studies in the literature (e.g., [20,24,26]). Furthermore, at least two different focus groups outside of Italy should be organized to extend the results regarding the software usability. This future work is required to evaluate the impact of the testers’ attitudes, backgrounds, and expertise on the results. At last, this comparison can be extended to assess other open source and commercial segmentation software tools to further extend the results available in the literature.

## Figures and Tables

**Table 1:** Patients’ characteristics.

Patient	Pathology	Image Properties
#1	osteonecrosis	surface collapse-geodes
#2	osteoarthritis	osteophytes-geodes
#3	osteoarthritis	several geodes
#4	osteoarthritis	several geodes
#5	osteoarthritis	big osteophyte-geodes
#6	osteoporotic fracture	decreased bone mass
#7	osteoarthritis	Osteophytes-geodes
#8	osteoarthritis	Osteophytes-geodes
#9	osteonecrosis	surface collapse-geodes
#10	osteoporotic fracture	decreased bone mass

**Table 2:** Signed Euclidean distance: average, absolute, and standard deviation between the reference (*G_D*) and test (*G_A*, *G_B*, and *G_C*) geometries. Values retrieved from *CloudCompare* (Figure 4 and Figure 5).

	Mimics (mm)			3D Slicer (mm)			Syngo.via Frontier (mm)		
Case	Average	Absolute	Std. Dev.	Average	Absolute	Std. Dev.	Average	Absolute	Std. Dev.
**#1**	−0.353	0.353	1.068	−0.800	0.800	1.024	−0.583	0.583	0.971
**#2**	−0.163	0.163	0.719	−0.156	0.156	0.686	−0.982	0.982	0.698
**#3**	−0.032	0.032	0.639	−0.037	0.037	0.556	−0.337	0.337	0.824
**#4**	−0.369	0.369	0.568	−0.237	0.237	0.524	−0.921	0.921	0.637
**#5**	−0.245	0.245	1.000	−0.198	0.198	0.794	−0.670	0.670	0.913
**#6**	−0.662	0.662	0.482	−0.482	0.482	0.652	−0.670	0.670	0.664
**#7**	−0.328	0.328	0.730	−0.168	0.168	0.782	−0.357	0.357	0.743
**#8**	−0.360	0.360	0.612	−0.317	0.317	0.559	−0.598	0.598	0.608
**Mean**	**−0.314**	**0.353**	**0.727**	**−0.299**	**0.299**	**0.697**	**−0.640**	**0.640**	**0.757**

**Table 3:** Femur heads’ measurements (H_D_: horizontal diameter and V_D_: vertical diameter) and means values for *G_A*, *G_B*, *G_C,* and *G_D* geometries.

	Reference (mm)			Mimics (mm)			3D Slicer (mm)			Syngo.via Frontier (mm)		
	V_D(G_D)_	H_D(G_D)_	Mean_G_D_	V_D(G_A)_	H_D(G_A)_	Mean_G_A_	V_D(G_B)_	H_D(G_B)_	Mean_G_B_	V_D(G_C)_	H_D(G_C)_	Mean_G_C_
**#1**	50.220	49.010	49.615	49.470	48.670	49.070	46.630	45.650	46.140	48.300	46.110	47.205
**#2**	53.530	50.150	51.840	52.890	51.880	52.385	54.830	52.080	53.455	51.350	49.500	50.425
**#3**	49.730	49.090	49.410	50.620	49.670	50.145	50.790	49.930	50.360	49.000	48.690	48.845
**#4**	44.050	43.620	43.835	44.700	42.120	43.410	44.560	43.080	43.820	42.450	41.400	41.925
**#5**	54.110	53.830	53.970	51.930	51.820	51.875	56.700	50.790	53.745	54.480	49.850	52.165
**#6**	43.550	43.080	43.315	44.620	39.970	42.295	43.770	39.540	41.655	43.820	41.460	42.640
**#7**	58.060	55.160	56.610	57.320	54.540	55.930	57.480	54.810	56.145	56.800	53.750	55.275
**#8**	45.980	40.080	43.030	43.960	40.320	42.140	42.630	39.560	41.095	41.620	40.450	41.035

**Table 4:** Signed and absolute deviations for horizontal and vertical femoral head diameters obtained from the three segmentation software.

	*Signed Deviation (mm)* *= Mean_G_X −_ Mean_G_D_*			*Absolute Deviation (mm)* *= |Signed Deviation|*			*Signed Percentage Deviation (%)* *= Signed Deviation/Mean_G_D_*			*Absolute Percentage Deviation (%)* *= Absolute Deviation/Mean_G_D_*		
	Mimics	3D Slicer	Syngo.via Frontier	Mimics	3D Slicer	Syngo.via Frontier	Mimics	3D Slicer	Syngo.via Frontier	Mimics	3D Slicer	Syngo.via Frontier
**#1**	−0.545	−3.475	−2.410	0.545	3.475	2.410	−1.1%	−7.0%	−4.9%	1.1%	7.0%	4.9%
**#2**	0.545	1.615	−1.415	0.545	1.615	1.415	1.1%	3.1%	−2.7%	1.1%	3.1%	2.7%
**#3**	0.735	0.950	−0.565	0.735	0.950	0.565	1.5%	1.9%	−1.1%	1.5%	1.9%	1.1%
**#4**	−0.425	−0.015	−1.910	0.425	0.015	1.910	−1.0%	0.0%	−4.4%	1.0%	0.0%	4.4%
**#5**	−2.095	−0.225	−1.805	2.095	0.225	1.805	−3.9%	−0.4%	−3.3%	3.9%	0.4%	3.3%
**#6**	−1.020	−1.660	−0.675	1.020	1.660	0.675	−2.4%	−3.8%	−1.6%	2.4%	3.8%	1.6%
**#7**	−0.680	−0.465	−1.335	0.680	0.465	1.335	−1.2%	−0.8%	−2.4%	1.2%	0.8%	2.4%
**#8**	−0.890	−1.935	−1.995	0.890	1.935	1.995	−2.1%	−4.5%	−4.6%	2.1%	4.5%	4.6%
**Mean**	**−0.547**	**−0.651**	**−1.514**	**0.867**	**1.293**	**1.514**	**−1.1%**	**−1.4%**	**−3.1%**	**1.8%**	**2.7%**	**3.1%**
**Std. Dev.**	**0.895**	**1.646**	**0.646**	**0.533**	**1.133**	**0.646**	**1.8%**	**3.4%**	**1.4%**	**1.0%**	**2.4%**	**1.4%**

**Table 5:** Usability evaluation of the three segmentation software tools.

Objective	Mimics	3D Slicer	Syngo.via Frontier	Weight	Mimics	3D Slicer	Syngo.via Frontier
1. Automatisation degree	High	Average	Low	10	1	2	3
2. Segmentation time	30 min	45 min	40 min	8	3	1	2
3. Training time	300 min	300 min	180 min	8	1	1	3
4. Cost	High	Freeware	Embedded in the CT	8	1	3	2
5. 3D visualisation	High	High	High	6	3	3	3
6. Supported Operative System (OS)	Windows—macOS—Linux	Windows—macOS—Linux	Windows	6	3	3	1
7. Potential extension (plugins)	No	No	No	4	1	1	1
**Total**				**50**	**1.80**	**2.00**	**2.28**

## Data Availability

Not applicable.
